# Supplementary material for: Pump-probe X-ray holographic imaging of laser-induced cavitation bubbles with femtosecond FEL pulses
Source: Nat Commun. 2021 Jun 8;12:3468. doi: 10.1038/s41467-021-23664-1 (PMC8187368; doi:10.1038/s41467-021-23664-1)
Supplement: Supplementary file 1 — Supplementary Information [file 41467_2021_23664_MOESM1_ESM.pdf]

Supplementary materials: Pump-probe X-ray holographic imaging of laser-induced cavitation bubbles with femtosecond FEL pulses

M. Vassholz[1], H. P. Hoeppe[1], J. Hagemann[2], J. M. Rosselló[3], M. Osterhoff[1], R. Mettin[3], T. Kurz[3], A. Schropp[2], F. Seiboth[2], C. G. Schroer[2,4], M. Scholz[5], J. Möller[5], J. Hallmann[5], U. Boesenberg[5], C. Kim[5], A. Zozulya[5], W. Lu[5], R. Shayduk[5], R. Schaffer[5], A. Madsen[5], and T. Salditt[1,*]

[1]Institut für Röntgenphysik, Georg-August-Universität Göttingen, Friedrich-Hund-Platz 1, 37077 Göttingen, Germany
[2]Deutsches Elektronen-Synchrotron (DESY), Notkestraße 85, 22607 Hamburg, Germany.
[3]Drittes Physikalisches Institut, Georg-August-Universität Göttingen, Friedrich-Hund-Platz 1, 37077 Göttingen, Germany
[4]Department Physik, Universität Hamburg, Luruper Chaussee 149, 22761 Hamburg, Germany
[5]European X-Ray Free-Electron Laser Facility, Holzkoppel 4, 22869 Schenefeld, Germany
[*]Corresponding author: tsaldit@gwdg.de

S1 High-speed optical imaging and plasma shape

Here, we show additional images of the optical high-speed camera, including images of the breakdown plasma luminescence. The optical camera observes the cavitation events from the side, perpendicular to the X-ray beam. An exemplary bubble cycle is depicted in Supplementary Fig. 1a. Note that this is the same cavitation event as shown in Fig. 2 of the main manuscript. As described in detail in the Methods section of the main manuscript, the maximum radius of expansion R_{max} of the cavitation bubble in combination with the lifetime τ measured with the microphone signal is used to determine the deposited bubble energy E_{B}. Furthermore, we can analyse the shape of the initial breakdown plasma, which is usually visible in the first frame of the high-speed video. We determine the number of plasma cores, each of them leading to the generation of a cavitation bubble and shockwave. Additionally, we can estimate an ellipticity factor $\epsilon_{\mathrm{opt}} = h/b$ of the breakdown plasma with the height h and width b of the plasma luminescence spot. Note that the image containing the plasma spark was exposed for the shutter opening time of 1.01 µs, during which the plasma already expanded to its maximum extent. The change of the plasma luminescence shape becomes obvious as ϵ_{opt} varies significantly already upon a shift of the optical camera trigger delay of multiples of 10 ns. In Supplementary Fig. 1b the first frame of a high-speed video is shown as a close-up, with two illustrated ellipses representing ϵ of the X-ray measurements and ϵ_{opt} of the optical camera for comparison. We observe different sizes and ellipticities in both measurements. Note that the X-ray hologram was taken with an ultra-fast flash at $\Delta t = 10$ ns whereas the optical image was taken with a shutter time of 1.01 µs. The ellipticity ϵ extracted from the X-ray data decreases with Δt. In the early expansion phase, the cavitation bubble is more elliptical (Supplementary Fig. 1c). However, we did not find a substantial correlation between ϵ_{opt} of the optical camera and the ellipticity ϵ of the X-ray data. This is not surprising not only in view of the different integration times, i.e. single pulse X-ray exposure versus >1 µs long exposure time of optical camera, but also the different contrast mechanisms. In the X-ray data, we probe the density profile of bubble and shockwave, whereas we primarily probe the plasma luminosity in the optical data.

As we induce cavitation in a sub-threshold regime of the seeding laser's irradiance, we mostly observe almost point-like or elliptical plasma cores which fluctuate in their position along the IR laser axis. In Supplementary Fig. 2a some examples of individual cavitation events are depicted. Supplementary Figures 2b and c show enlarged images of two cavitation events. The latter is a rather rare event (in the sub-threshold regime) with multiple elliptical plasma cores and is not further processed in the X-ray analysis workflow (vetoed out). Note that in this run, the timing of the 1.01 µs optical shutter was chosen such that the camera is illuminated until 260(25) ns after optical breakdown. The outer limit of the bubble thus corresponds to its maximum expanse at a time of about 260 ns.

In Supplementary Fig. 1d–f we show a cavitation event with two breakdown plasma cores. The double event is visible in both measurements, in the side view of the optical camera and in the 2d phase reconstructed hologram (Alternating

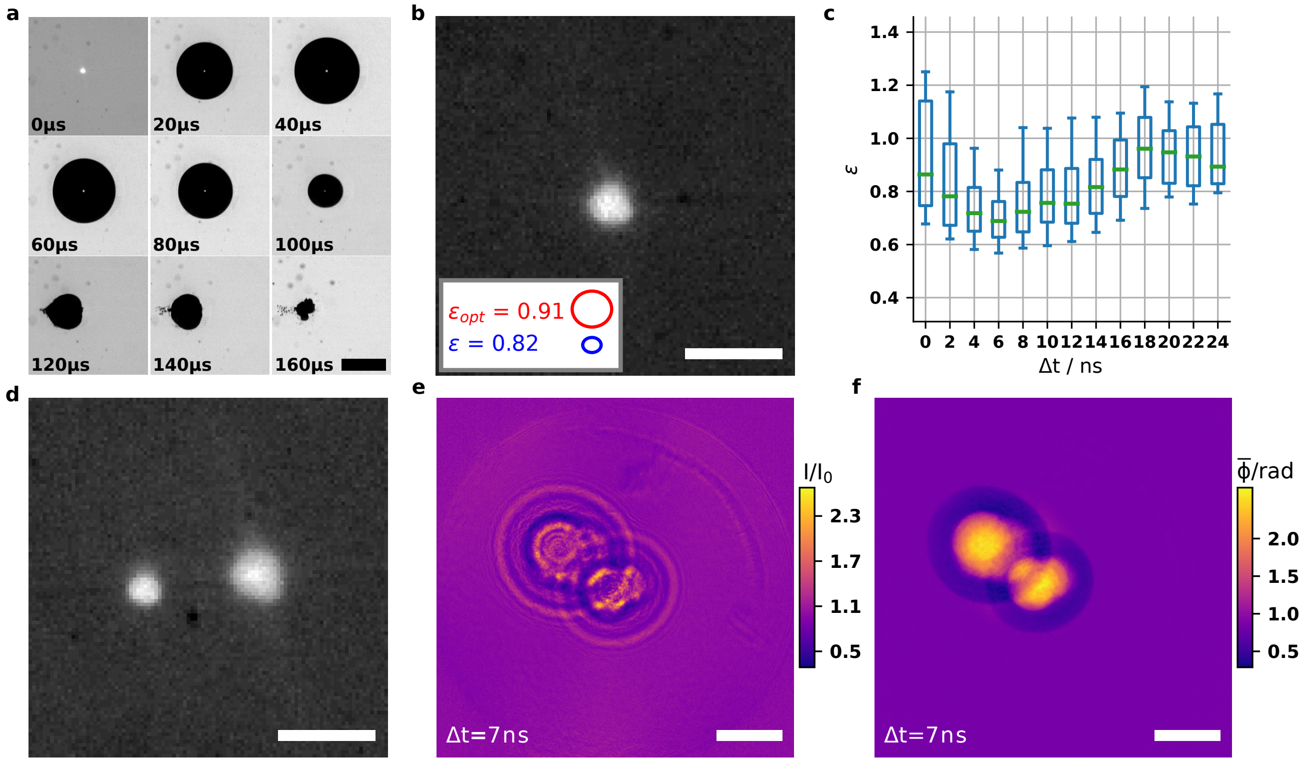

Supplementary Fig. 1: Observations of the plasma shape. **a**, Optical high-speed measurements of a full bubble cycle. The first frame is synchronized to the X-ray pulse with a time delay of $\Delta t = 7\,\mathrm{ns}$ after the IR-pump pulse. **b**, Enlarged view of the plasma luminescence, with indicated ellipticity and scale of the optical (red) and X-ray (blue) measurements. The two ellipses are drawn to scale. The same cavitation event is shown as in Fig. 2. **c**, Box-whisker plot of the ellipticity ϵ from the X-ray measurements, with respect to 2 ns bins of Δt (green line, median; the box contains 50 % of the data; whiskers show the 10th and 90th percentile). **d**, single frame of optical high-speed measurement featuring a double-breakdown event. **e**, Normalized intensity I/I_0 of the X-ray hologram of the event in (d). Note, that the observation direction of the optical and X-ray imaging are perpendicular. **f**, AP phase reconstruction of the event in (d). Scale bars: 750 µm (a), 200 µm (b, d), 25 µm (e, f).

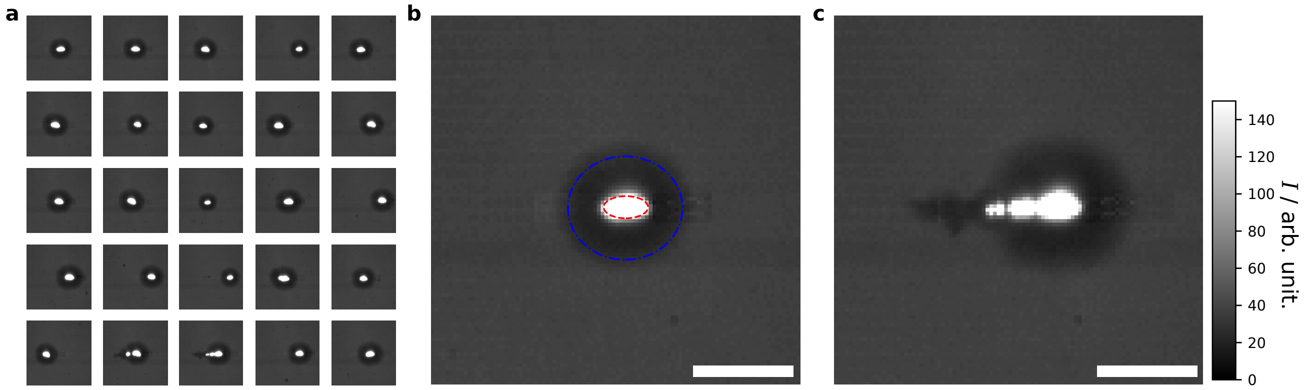

Supplementary Fig. 2: Optical observations of the plasma and cavitation bubble. **a**, Exemplary overview of individual cavitation events. The position of the plasma core fluctuates along the laser beam axis. **b**, Enlarged view of a cavitation event with an ellipsoidal plasma core. The early state of the expanding bubble is visible as the dark region surrounding the plasma spark. The bubble-water interface is blurred due to its motion. We extract semi-major and -minor axes of $a_p = 44\,\mu m$ and $b_p = 22\,\mu m$ for the plasma core (red) and $a_b = 114\,\mu m$ and $b_b = 103\,\mu m$ for the expanding bubble (blue). **c**, Cavitation event with multiple plasma cores. Such events can be identified by the optical measurements and are vetoed out in the X-ray analysis workflow. In this measurement, the timing of the optical shutter was chosen such that the camera illuminates until 260(25) ns after optical breakdown. Scale bars: 200 µm.

Projections [AP] phase retrieval [1]). Such events, including even more chaotic scenarios as shown in Supplementary Fig. 11, are not suited to compute $\phi(R)$ with the radial symmetric phase retrieval approach RFP, however can be reconstructed with the 2d approach. For instance, in Supplementary Fig. 1f one clearly recognizes the two compression waves emanating from the plasma cores. Events like this could be further analyzed in terms of bubble and shock densities of nascent or even crossing shock waves from different breakdown positions. However in the presented work, such events have been excluded from the analysis.

S2 Spatial resolution and scalability

We briefly discuss the spatial resolution and its scalability for the method of single pulse holography using cone-beam geometry. Importantly, X-ray holography is not subject to the resolution restrictions known for optical imaging of cavitation bubbles. In fact, as stable seeding of cavitation bubbles requires substantial distances from the cuvette wall optical imaging with high numerical aperture objectives is impeded, and resolutions in the range 2–10 µm are already quite exceptional. This is not the case for X-ray imaging in cone-beam geometry, which in principle can achieve the resolution given by the focal spot size. The present experiment was designed for sub-100 nm spot size (CRL focus), and a well matched geometrical magnification of 66, resulting in an effective pixel size of 98 nm. However, different resolution deteriorating factors have to be taken into account, including possible aberrations of the incoming beam, bandwidth of the XFEL radiation, detector PSF, etc.

To obtain an estimation on the resolution of the presented method, we analyze the interface profile of a stably floating bubble. This seems a better choice than a cavitation bubble, since the out-of-equilibrium nature of a cavitation bubble could result in a broadened interface between the gas and aqueous phase. Supplementary Fig. 3a, b show the X-ray hologram of a floating bubble and the angularly averaged intensity, respectively. The highest spatial frequency encoded in the hologram can be estimated by the maximum scattering angle of the interface, or more precisely the angular range of fringe visibility. Supplementary Fig. 3b shows fringes extending over an angular range of 0.14 mrad, corresponding to a half-width-at-half-maximum (HWHM) resolution of 3.8 px, or 370 nm. Note that the possible resolution given by the entire illuminated detector area and the numerical aperture of the CRLs with a theoretical focal spot size of 78 nm (FWHM) is in principle much higher.

To quantify the influence of the phase retrieval on the resolution, we analyze line profiles of the reconstructed phase. Supplementary Fig. 3c shows the reconstructed phase (2d projected phase) of the equilibrium bubble, as reconstructed by the AP algorithm. Profiles along the horizontal and vertical direction as well as radially averaged profiles are then used for a regularized inverse Abel transform to obtain the reconstructed phase $\phi(r)$ (3d phase), which is proportional to the electron density. A modified error function with width σ is fitted to the curve to obtain the interfacial width. We find 500(40) nm for the horizontal profile, 480(60) nm for the vertical profile and 620(20) nm for the radial average. Furthermore, we calculate the edge steepness from the density profile, obtained by the RFP reconstruction algorithm. Here, the interfacial profile $\phi(r)$, fitted to an error function, results in a HWHM of 460(20) nm. Note that all given

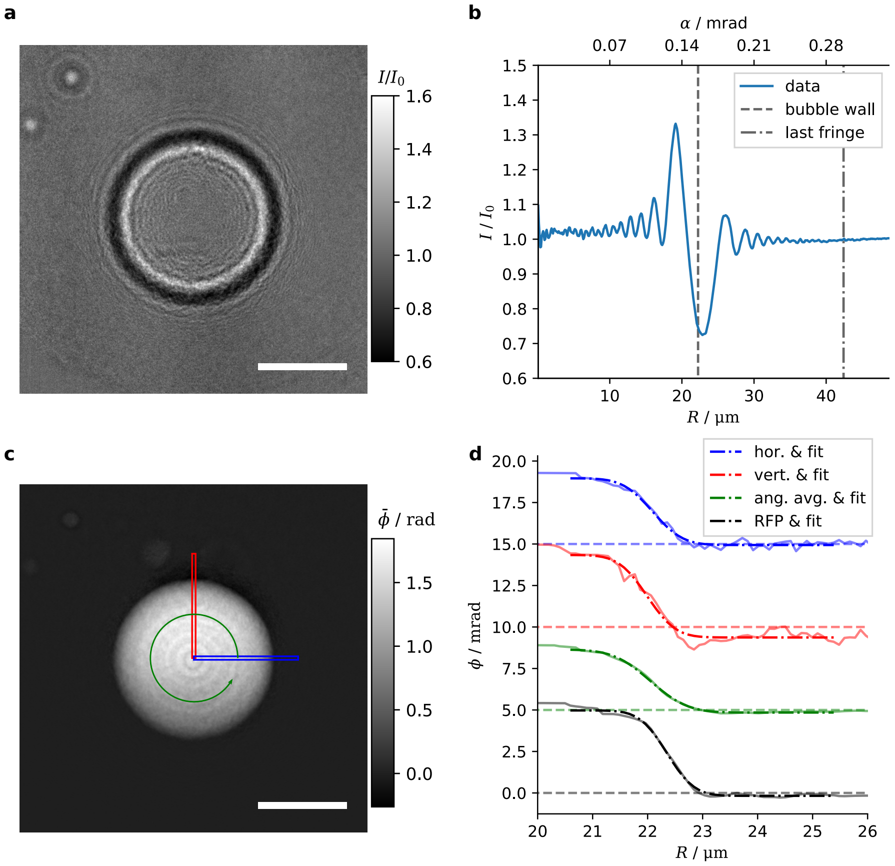

Supplementary Fig. 3: Resolution estimation: **a**, Normalized intensity I/I_0 of the X-ray hologram of a freely floating bubble, i.e. no cavitation bubble. **b**, Angular average of the intensity of the X-ray hologram (a). The upper abscissa shows the diffraction angle α on the detector. The angle of maximum Fresnel diffraction (fringe visibility), i.e. the range spanned between the bubble wall and the outermost fringe is given by $\alpha_{\mathrm{max}} \approx 0.14$ mrad, corresponding to a resolution of about 370 nm (HWHM). **c**, AP reconstruction of the projected phase $\bar{\phi}$ of the floating bubble (a). In the marked regions line profiles have been extracted for further analysis of the resolution. **d**, Reconstructed radial (3d) phase profiles $\phi(r)$ as a function of the radius R. The HWHM resolution is determined from an error function fitted to the data. The blue, red and green curves show the radial 3d phase $\phi(r)$ of the line profiles from the AP reconstruction in (c). For better visibility, the curves are offset by 5 mrad. The dashed lines indicate zero phase shift for each color. The obtained HWHM resolution is 500(40) nm (horizontal, blue), 480(60) nm (vertical, red), and 620(20) nm (angular average, green). The black curve is obtained from the phase retrieved by the RFP algorithm. The HWHM resolution of the RFP phase is 460(20) nm. Scale bars: 25 µm corresponding to a detection angle of 0.18 mrad in (a); 25 µm in (c).

values represent the HWHM of the edge, given by HWHM $= 1.17\sigma$, with σ the RMS-width of the error function. Importantly, the achieved resolution is sufficient to probe the interface profiles of the cavitation bubbles. The cavitation event shown in Fig. 2 of the main manuscript, for example, exhibits an edge width of 1270(10) nm for the bubble boundary and 690(20) nm (HWHM from RFP) for the shock front, well in excess of the resolution limit. We can take this as an indication that the interfacial width of cavitation bubbles is intrinsically broader than the equilibrium gas-water interface.

Nevertheless, the theoretically achievable resolution of sub-100-nm is not reached. We have identified the spectral bandwidth of the SASE radiation of the XFEL with approximately 60 eV (FWHM) to be a major cause for a focus broadening and thus a degradation of the resolution. CRLs are chromatic optics, i.e. different photon energies are focused with a different focal length. The focal length f is proportional to the inverse of the decrement δ of the refractive index $f \propto \delta^{-1}$ [2] and the decrement scales with $\delta \propto E^{-2}$. Hence, for small variations ΔE in the photon energy we get a deviation Δf of the focal length of approximately $\Delta f/f \approx 2\Delta E/E$. If we approximate the spectral bandwidth of the XFEL pulses to be on the order of $\Delta E/E \approx 4 \times 10^{-3}$ (FWHM), we get for $f = 298$ mm a deviation in the focal length of $\Delta f \approx 2.4$ mm (FWHM). This value is almost 15-times the Rayleigh length of 160 µm. The beam diameter at $\Delta f/2 \approx 1.2$ mm defocus is already larger than $\gtrsim 600$ nm (FWHM). The superposition of the different longitudinal modes of the XFEL radiation thus results in a substantial broadening of the X-ray focus and consequently to a degradation of the holographic resolution. The approximated focal width of $\gtrsim 600$ nm (FWHM) is in good agreement with the resolution obtained from the maximum scattering angle of the holograms of 370 nm (HWHM).

Next, we briefly comment on scalability. Cone-beam X-ray holographic imaging has been demonstrated at resolutions down to 25 nm [3], based on waveguide-filtered synchrotron radiation. For the present single pulse holographic imaging experiment, scaling up the resolution would require higher longitudinal coherence or an achromatic optic, to achieve a truely diffraction-limited spot size in the sub-100 nm range. To this end, either the longitudinal coherence of the beamline needs to be improved by e.g. seeded SASE radiation or achromatic optics like X-ray waveguides, with even higher numerical apertures, could be exploited.

Last, we briefly compare the present spatial resolution to optical imaging of cavitation bubbles. As cavitation is usually investigated in bulk water, i.e. a cuvette with significant extent, long-distance objectives are used for imaging. As a benchmark example we can refer to [4], where the detailed shape of a large plasma cone of laser seeded cavitation bubble is resolved at a working distance of 35 mm, and with a resolution stated as 4 µm. Stan et. al [5] imaged XFEL-induced explosions of a water jet with optical imaging. In this special case of an experiment without the need for a water-filled cuvette, they obtained a resolution of 780 nm (half cycle) for a test pattern and ∼1 µm for the water jet. To our knowledge, however, such resolution values were not yet achieved in imaging for cavitation in bulk water. Furthermore, in contrast to the present approach, it is impossible for optical cavitation imaging to scale the resolution up. More importantly, optical contrast does not allow for a direct measurement of the shockwave density close to a cavitation nucleus, let alone a quantitative pressure profile.

For visual appreciation of the high spatial sampling in the present x-ray imaging approach, we include Supplementary Fig. 4, showing two enlarged images of early states of laser-induced cavitation events. Both the flat-field corrected X-ray hologram as well as the AP reconstructed phase shift $\bar{\phi}$ is shown in each image. Sub-micrometer scale density fluctuations are visible both in the X-ray hologram as well in the reconstructed phase. These can be attributed to fluctuations in the initial breakdown plasma which still persist after plasma growth.

S3 Influence of ellipticity

In the present experimental setting, no lateral X-ray view of the cavitation bubble was recorded. While the high-speed optical camera can help to control ellipticity ϵ and to veto out strongly asymmetric cavitation events, e.g. due to formation of multiple plasmas (see Supplementary Fig. 1), the illumination times of the optical camera are too long to match the X-ray acquisitions. They hence can only inform on bubble shapes at later times, or more precisely, show shapes which must be regarded as a time integral. For this reason, the assumption of a spherical or elliptical shape when reconstructing a radial profile of the cavitation bubble, has to be critically questioned. Non-spherical shapes of cavitation bubbles have been investigated by methods of linear stability analysis, showing that the growth process is stable [6], with perturbations subject to either damped or over-damped relaxation. For viscous fluids the latter is true and the perturbation amplitude tends to zero as the bubble grows [6]. At the same time, the initial conditions for laser seeded bubbles result in axial-symmetric but not spherical-symmetric initial conditions. We therefore must expect elliptical or even pear-formed bubble deformations at the early times, see Supplementary Fig. 1, and the discussion in the main manuscript regarding the constraint of positive electron density in the bubble center. Hence, while one observes a spherical shape with bubble radius R_0 from the projection image (or equivalently the hologram), the actual radius of curvature may be higher $R \geq R_0$. In the simplest case we would expect an elliptical half axis along the optical axis z with $R_z = R_0/\epsilon$, but in the beginning, pear-like shapes are also likely. Importantly, both elliptical and non-elliptical (but differentiable) shape deformations can be treated in the same manner in form of a Taylor expansion

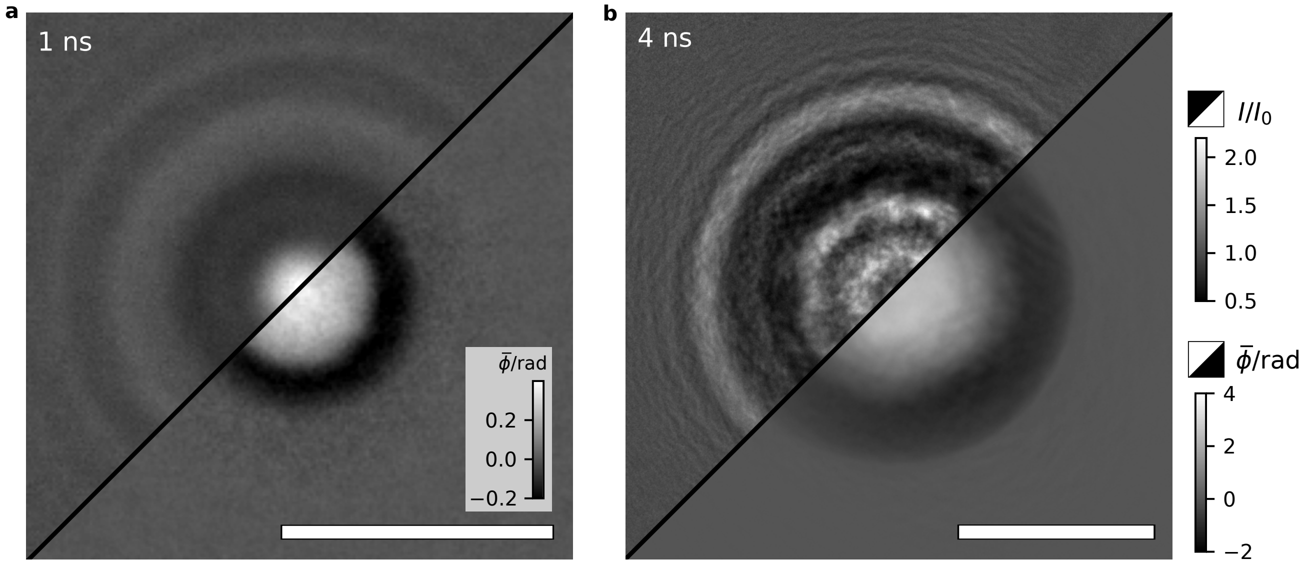

Supplementary Fig. 4: X-ray holograms and retrieved phase image. Cavitation events probed at **a**, $\Delta t = 1\,\mathrm{ns}$ and **b**, $\Delta t = 4\,\mathrm{ns}$ respectively, during optical breakdown and plasma expansion. The upper left part of the images shows the normalized intensity I/I_0 of the X-ray hologram. The bottom right part shows the X-ray phase shift $\bar{\phi}$ in the sample plane, obtained by AP phase reconstruction. Scale bars: 10 µm.

around the spherical (or even the elliptical) case. Since, however, the projection of an ellipsoid is again an ellipse, an ellipsoidal deformation results in a projection profile $P(x)$ which is scaled by a prefactor, but preserves its functional form, see the schematics in Supplementary Fig. 5. In the same manner, a spherically bulged slab of radius R and thickness d with, for example, a flat radial profile (hat profile), projects to a profile

$$P(x) = \begin{cases} 2\left(\sqrt{(R+d)^2 - x^2} - \sqrt{R^2 - x^2}\right) & , x \le R \\ 2\sqrt{(R+d)^2 - x^2} & , R < x \le R + d \\ 0 & , x > R + d \ . \end{cases}$$

Written in unitless coordinates $x' = (x - R)/d$ and $R' = R/d$, and expanding for $R' \ll 1$, the projected profile becomes [7, 8]

$$P(x') \simeq \begin{cases} 2\left(\sqrt{1 - x'} - \sqrt{-x'}\right) & , -R' \le x' \le 0 \\ 2\sqrt{1 - x'} & , 0 < x' \le 1 \\ 0 & , x' > 1 \ . \end{cases}$$

Hence an inverse Abel transform, falsely assuming radial symmetry and not accounting for $\epsilon \neq 1$ would still yield the correct flat hat profile of the slab. Since arbitrary profiles can be approximated by concentric shells (each with a hat function), the shapes of the projection profiles are preserved unless the deformations become excessively large or non-differentiable. Supplementary Fig. 6 presents numerical solutions to corroborate the conclusions drawn from the analytical slab model. Again, we see that the hat profile reflecting a flat density profile of compressed water in the shock wave around a distorted gas bubble projects to the same functional form $P(x)$, when restricting the range of x to the range of the projected shock wave. More relevant still, is the radial density profile $\rho(r)$ computed by the inverse Abel transform from $P(x)$, which clearly reconstructs a flat hat profile for all cases shown. Altogether, this confirms our conclusions in the main text. The deviations of the experimental shock waves from the fluid dynamical model predictions, as shown in in Fig. 5c–e of the main manuscript, cannot be explained by parameter choices regarding the radius of curvature, or equivalently ϵ or even more general shape deformations along the optical axis. Instead the deviations, and in particular the inversion of the pressure slope has to be attributed to shortcomings of the model, which were previously not observed, since the density and pressure profile could not be properly accessed. At the same time, we stress that in the relatively simple scenario treated here, the density of the shockwave does not vary along the directions parallel to the bubble surface, which could be expected for deformed bubbles. In the main text, we therefore suggest experimental geometries which are able to probe also such effects.

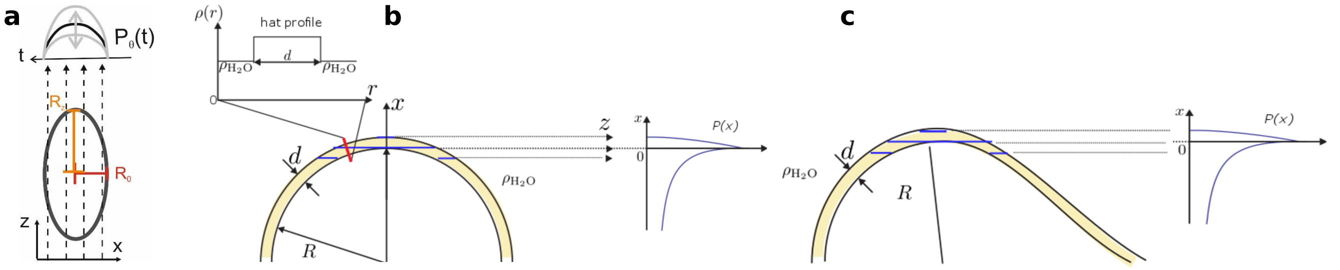

Supplementary Fig. 5: Projection of ellipsoids and shapes with local deviation of the radius of curvature. **a**, Schematic of the projection of an elliptically deformed bubble. While one observes a spherical shape with bubble radius R_0 from the projection image (or equivalently the hologram), the actual elliptical half axis along the optical axis z is $R_z > R_0$. This results in a different prefactor of the projection profile $P(t)$. The functional form of $P(t)$ is preserved. **b, c**, Projection of a spherically bulged slab of thickness d, and radius R. Up to quadratic order in d/R, the shape of the projection profile $P(x)$ in the range $R < x < R + d$ remains constant, i.e. $P(x)$ is only scaled by a prefactor proportional to R. Hence small deviations around the assumed spherical shape would not affect the shape of the radial profile obtained from the inverse Abel transform. This can be shown analytically for a hat profile, and by linearity translates to general analytical radial profiles.

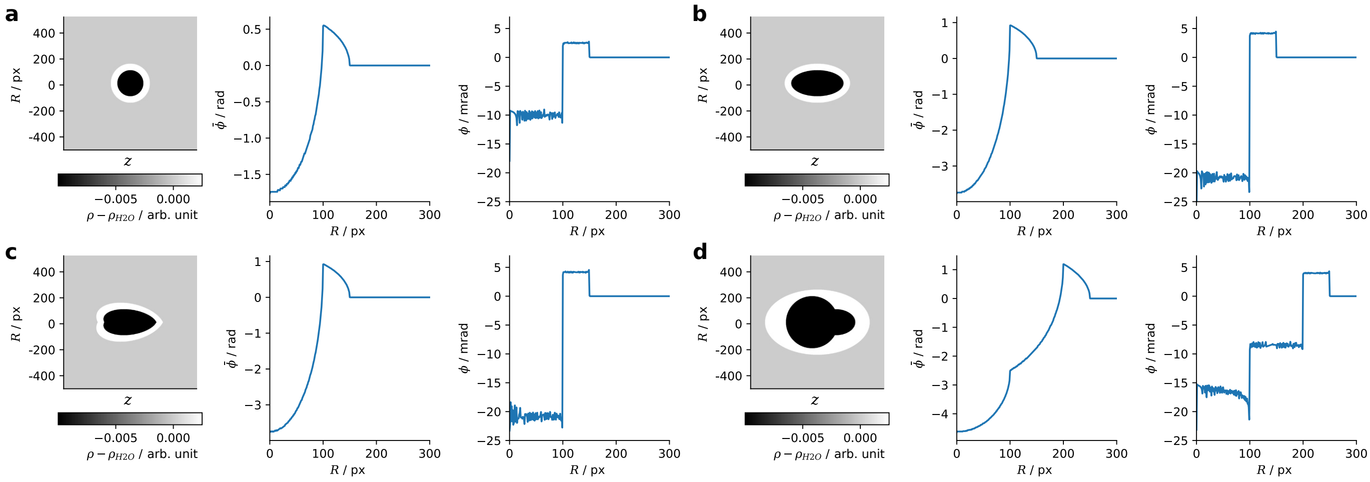

Supplementary Fig. 6: Numerical calculation of radial profiles from bubble phantoms with shape deformations. For each case the phantom (2d) is shown on the left with X-ray optical parameters set to the experimental values, for shockwave (white), and bubble (black), immersed in bulk water (gray). z denotes the direction of propagation of the X-rays. The center shows the corresponding projected phase profile and on the right the radial profile extracted from the inverse Abel transformation is shown, i.e. falsely assuming spherical symmetry. The following cases, including unrealistically high shape distortions, all lead to the same and correct functional form for the radial density profile of the shockwave, here assumed as a flat profile: **a**, Perfect spherical symmetry for reference, **b**, elliptical distorted bubble, **c**, 'bullet' shape, and **d**, bubble with a bud.

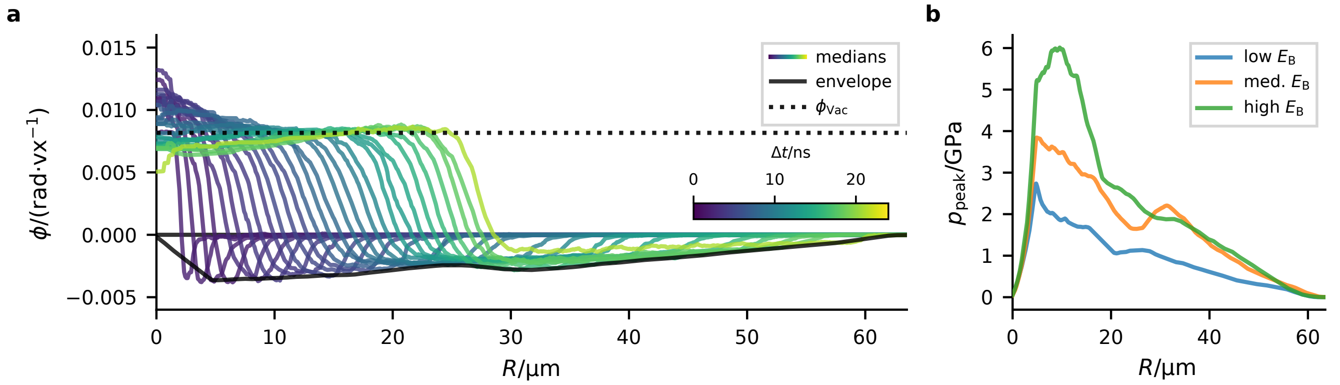

Supplementary Fig. 7: Cavitation dynamics with ellipticity correction. **a**, Median of the phase profiles for different ranges of the bubble radius R_B, showing how the median phase evolves with time ($E_B = 66$–$130\,\mu J$). In comparison to Fig. 4c, the phase profiles have been corrected with the bubbles ellipticity ϵ before calculation of the median. The color represents the median of the time delay Δt. The (smoothed) envelope of the shockwave's phase shift (black) is used to calculate the shockwave's pressure as a function of the distance to the bubble center R. **b**, Peak pressure p_{peak} as a function of the distance to the bubble center R, obtained from the envelope of the ellipticity corrected shockwave's phase shift for energy ranges E_B between 7–66 µJ (low E_B), 66–130 µJ (med. E_B) and 130–250 µJ (high E_B).

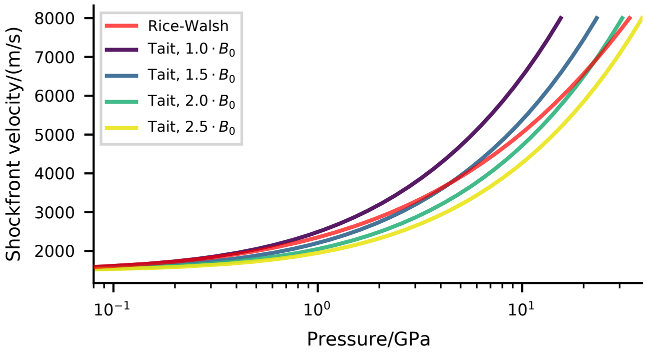

Supplementary Fig. 8: Equations of state. Comparison of the Rice-Walsh equation of state [11] to the Tait equation of state with different constants B. The Tait equation of state with $B = B_0$ approximates the Rice-Walsh equation of state well for pressures below ~2.5 GPa [9]. Consequently, the shockfront velocity is overestimated by the Tait equation for higher pressures. We compensate this effect by adjusting the constant B of the Tait equation to $2B_0$.

S4 Modification of the Tait equation

The Tait equation fits experimental data for pressure values up to 2.5 GPa [9]. For higher pressures however, the shockwave velocity u_s is overestimated [10]. This leads to an overestimation of the simulated shockwave radii $R_{\text{SW}}(\Delta t)$. Supplementary Fig. 8 shows $u_s(p_s)$ calculated using the Tait equation for different values of B compared to the Rice-Walsh equation of state, with validity of up to ~25 GPa [11]. As the Rice-Walsh equation of state cannot easily be incorporated into the Gilmore model equation [4], we compensate for this deviation, by treating B as an effective parameter in the Tait equation. Increasing B effectively decreases the shockwave velocity $u_s(p_s)$ for high pressures. It can be interpreted as an increase in the effective bulk modulus of water for high pressures. With an adjustment of B to $2B_0$ ($B_0 = 314\,\text{MPa}$ [12]), we underestimate $u_s(p)$ for low values of p, but get a better agreement with the Rice-Walsh data for higher pressures. As our simulations quickly grow to high pressures, we obtain better agreement of $R_{\text{SW}}(\Delta t)$ with the data for $B = 2B_0$ (c.f. Fig. 5a and Fig. 5a–c). Note that the adjustment of B has strong impact on the trajectory $R_{\text{SW}}(\Delta t)$, whereas $R_B(\Delta t)$ is only slightly changed.

S5 Imaging of the bubble collapse

So far, we have focused on imaging and analysis of the optical breakdown, shockwave emission and the early states of bubble expansion. However, the presented experimental technique is in principle also suited to image the bubble collapse. The present timing scheme does not facilitate a synchronization of the FEL pulse and the bubble collapse, hence does not allow a systematic measurement of the bubble collapse. We find a mean bubble lifetime $\tau = 120\,\mu s$ with a standard deviation of $\sigma = 24\,\mu s$ [13]. With the 10 Hz repetition rate of the XFEL and a 25 % seeding rate of the

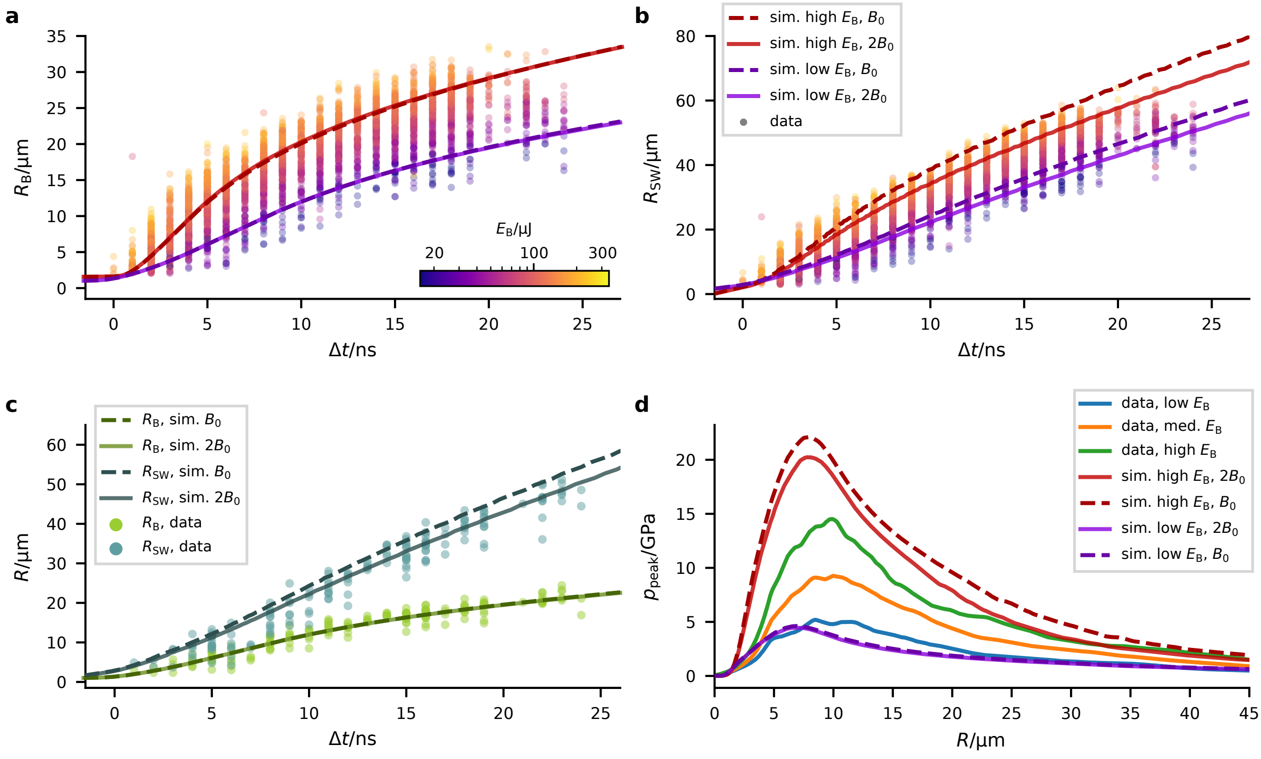

Supplementary Fig. 9: Simulations. Simulated trajectories of bubble wall radius R_B **(a)** and shockwave radius R_{SW} **(b)**. The data is shown as scatter dots for comparison. The color scales with the bubble's energy (shared colorbar, logarithmic scale). The simulation was run with 2 different sets of parameters to fit to lower energetic cavitation events ($E_B \approx 20$–$33\,\mu$J) and to fit higher energetic cavitation events ($E_B \approx 111$–$130\,\mu$J), as well as with two different values for the constant B (see main text). Whereas the bubble wall trajectories fit the data well for both values of B, the shockwave trajectory overestimates R_{SW} of the data for $B = B_0$. The radius of maximal expansion of the simulations yields a bubble energy of $22\,\mu$J for the low E_B and $91\,\mu$J for the high E_B simulation. **c**, Trajectory of R_B and R_{SW} for the low E_B simulation. Here, only experimental data in the energy range of $E_B \approx 20$–$33\,\mu$J is shown. **d**, peak shockwave pressure p_{peak} in a distance R to the bubble center for three energy ranges of the experimental data (c.f. Fig. 4d) and the simulations. The pressure profiles of the simulations have been convolved with a Gaussian function (500 nm FWHM) before calculation of the peak pressure p_{peak}.

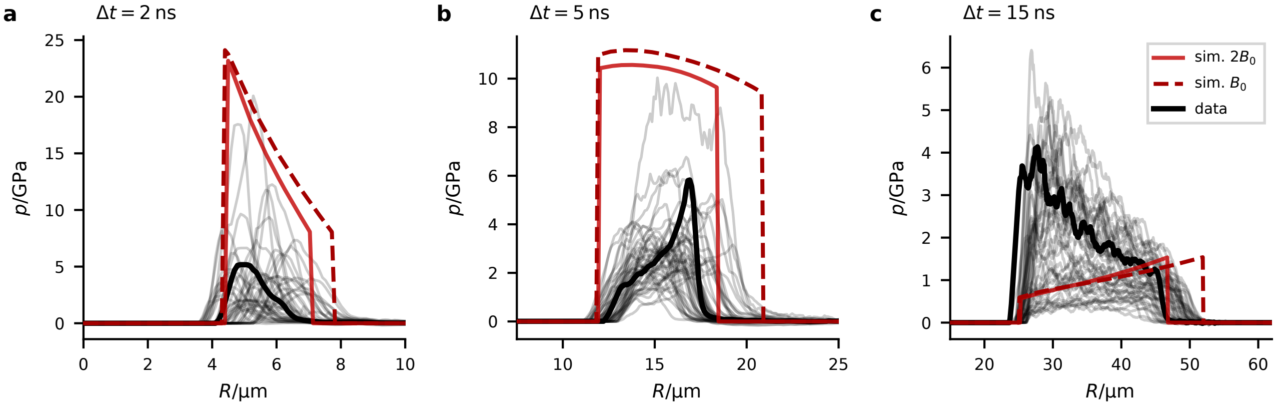

Supplementary Fig. 10: Pressure distribution with compensated ellipticity. **a**–**c**, comparison of the measured, ellipticity corrected, shockwave's pressure profile $p(R)$ with the simulated pressure profiles (high E_B simulation) for three different time delays Δt as indicated in the top left corner. The simulation is shown for both values of B. The bold black curve shows the pressure profiles from Fig. 3, the gray curves are a selection of pressure profiles within the energy range $E_B \approx 111$–$130\,\mu$J. All experimental pressure distributions have been corrected with the calculated ellipticity factor ϵ.

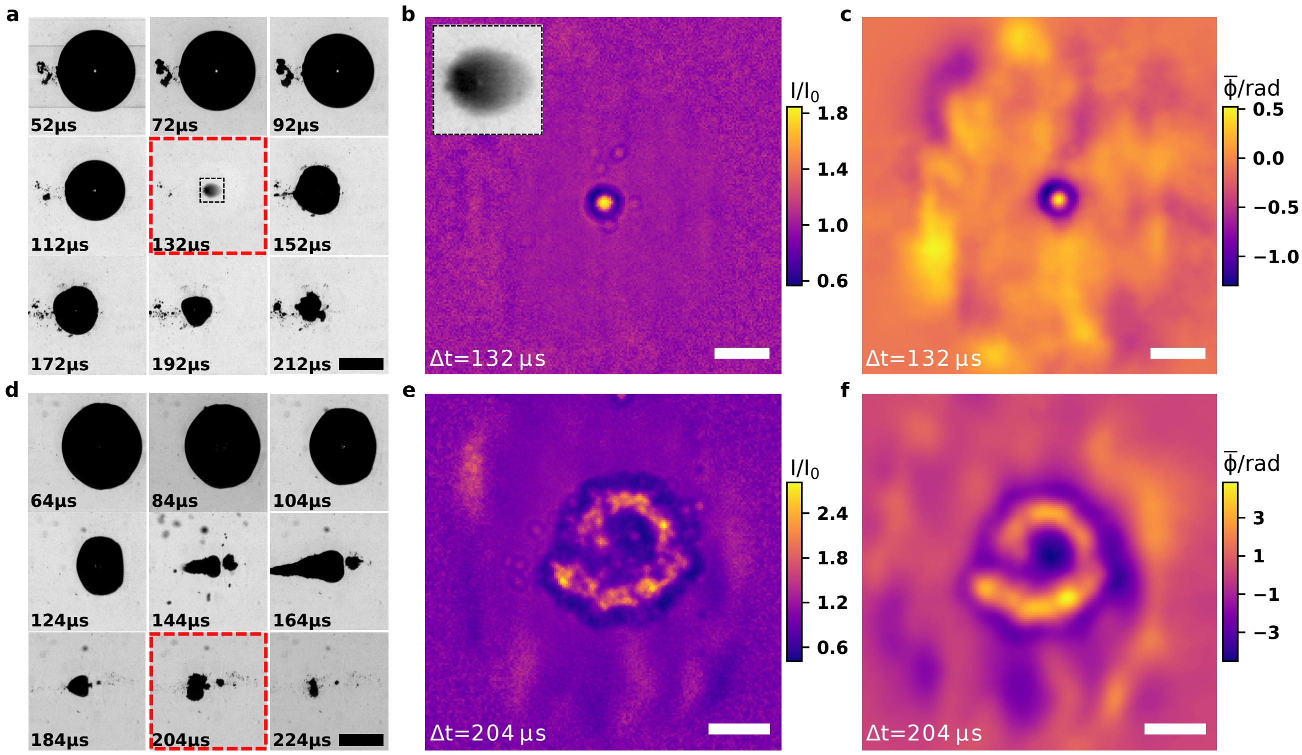

Supplementary Fig. 11: Capturing the bubble collapse. **a**, Optical high-speed measurement of a bubble cycle. The 5th frame was synchronized to the X-ray pulse, with a time delay of $\Delta t = 132\,\mu s$ to the IR-pump pulse. **b**, Normalized intensity I/I_0 of the X-ray hologram of the collapsing bubble in parallel beam geometry. The 5th frame of the optical images is shown at the same scale for comparison in the inset. **c**, Paganin-type phase reconstruction of the projected phase $\bar{\phi}$ of the collapsing bubble. **d**, optical high-speed measurement of a bubble cycle. The 8th frame was synchronized to the X-ray pulse, with $\Delta t = 204\,\mu s$. **e**, hologram of the chaotic bubble rebound after the collapse, of the event shown in (d). **f**, Paganin-type phase reconstruction of the chaotic bubble rebound. Scale bars: 750 µm (a, d), 200 µm (b, c, e, f).

cavitation bubbles we might image the bubble collapse within a time window of 5 ns by chance, once in 1.5 hours. In Supplementary Fig. 11a–c a cavitation event is depicted where we observed the bubble close to the collapse. Supplementary Fig. 11a shows the optical high-speed video, Supplementary Fig. 11b the near-field hologram recorded with the parallel X-ray beam (without nanofocusing CRLs) and Supplementary Fig. 11c the phase retrieval using the single materials approach [14]. The X-rays capture the collapsing bubble within the exposure of the fourth frame of Supplementary Fig. 11a, with a time delay $\Delta t = 132\,\mu s$ after seeding. From the bubble lifetime and the delay between seeding laser and FEL pulse, we estimate that the bubble was probed ∼300 ns before the collapse. The parallel beam geometry was chosen in this run to illuminate a larger field of view, so that also large bubbles at such high time delays and collapse rebounds fit into the field of view. Supplementary Fig. 11d–f shows a high-energy cavitation event with a high elongation and jetting during the collapse, due to multiple plasma sparks [15]. The video of the HS camera is shown in Supplementary Fig. 11d with the X-ray flash during the 8th frame at 204 µs after seeding. Supplementary Fig. 11e depicts the X-ray hologram and Supplementary Fig. 11f the phase retrieval using [14]. The hologram was recorded after the second collapse during the second rebound. Supplementary Fig. 11f clearly shows the torus-like bubble shape with the liquid region in the center, originating from the jet. Furthermore, a liquid bridge can be recognized traversing the torus sideways. This demonstrates that such recordings can image details of non-spherical bubble collapses that are otherwise obscured to optical imaging. The parallel beam geometry comes at the cost of lower resolution and lower quality of the phase retrieval. Nevertheless, we emphasize the versatility of the method and the experimental setup, enabling also single-shot imaging of millimeter-scale involved structures. However, high-resolution phase contrast images are necessary to image the fine details during the bubble collapse. For this reason, we plan to synchronize the bubble collapse with the FEL pulse by acoustic trapping of the laser-induced cavitation bubbles. The ultrasonic field drives the bubble periodically, with frequencies locked to the FEL's repetition rate, so that the X-ray flash comes at a fixed point of time in the life cycle of the bubble. In this case, the X-ray flash can be synchronized to the collapse of the bubble with a tunable delay, to measure the collapse dynamics with high-resolution holography setup and the presented method.

References

[1] J. Hagemann, M. Töpperwien, and T. Salditt. Phase retrieval for near-field X-ray imaging beyond linearisation or compact support. *Appl. Phys. Lett.*, **113**(4), 2018.

[2] C. G. Schroer, B. Lengeler, B. Benner, T. F. Guenzler, M. Kuhlmann, A. S. Simionovici, S. Bohic, M. Drakopoulos, A. A. Snigirev, I. Snigireva, and W. H. Schroeder. Microbeam production using compound refractive lenses: beam characterization and applications. In *X-Ray Micro- and Nano-Focusing: Applications and Techniques II*, volume 4499, pages 52–63. International Society for Optics and Photonics, 2001. doi: 10.1117/12.450222.

[3] M. Bartels, M. Krenkel, J. Haber, R. N. Wilke, and T. Salditt. X-Ray Holographic Imaging of Hydrated Biological Cells in Solution. *Phys. Rev. Lett.*, **114**:048103, 2015.

[4] A. Vogel, S. Busch, and U. Parlitz. Shock wave emission and cavitation bubble generation by picosecond and nanosecond optical breakdown in water. *J. Acoust. Soc. Am.*, **100**(1):148–165, 1996.

[5] C. A. Stan, D. Milathianaki, H. Laksmono, R. G. Sierra, T. A. McQueen, M. Messerschmidt, G. J. Williams, J. E. Koglin, T. J. Lane, M. J. Hayes, S. A. H. Guillet, M. Liang, A. L. Aquila, P. R. Willmott, J. S. Robinson, K. L. Gumerlock, S. Botha, K. Nass, I. Schlichting, R. L. Shoeman, H. A. Stone, and S. Boutet. Liquid explosions induced by X-ray laser pulses. *Nat. Phys.*, **12**(10):966–971, 2016. doi: 10.1038/nphys3779.

[6] A. Prosperetti and G. Seminara. Linear stability of a growing or collapsing bubble in a slightly viscous liquid. *The Physics of Fluids*, **21**(9):1465–1470, 1978. doi: 10.1063/1.862408.

[7] A. Beerlink, M. Mell, M. Tolkiehn, and T. Salditt. Hard x-ray phase contrast imaging of black lipid membranes. *Applied Physics Letters*, **95**(20):203703, 2009. doi: 10.1063/1.3263946.

[8] M. Mell. Phase Contrast Imaging of Lipid Bilayer Model Membranes using Hard X-Rays. Diplomarbeit, Georg-August-Universität, Göttingen, Germany, 2009.

[9] J. M. Richardson, A. B. Arons, and R. R. Halverson. Hydrodynamic properties of sea water at the front of a shock wave. *J. Chem. Phys.*, **15**(11):785–794, 1947. doi: 10.1063/1.1746334.

[10] A. Vogel, K. Nahen, D. Theisen, and J. Noack. Plasma formation in water by picosecond and nanosecond Nd:YAG laser pulses. I. Optical breakdown at threshold and superthreshold irradiance. *IEEE J. Sel. Top. Quantum Electron.*, **2**(4):847–860, 1996. doi: 10.1109/2944.577307.

[11] M. H. Rice and J. M. Walsh. Equation of State of Water to 250 Kilobars. *J. Chem. Phys.*, **26**(4):824–830, 1957. doi: 10.1063/1.1743415.

[12] S. Ridah. Shock waves in water. *J. Appl. Phys.*, **64**(1):152–158, 1988. doi: 10.1063/1.341448.

[13] M. Osterhoff, M. Vassholz, H. P. Hoeppe, J. M. Rosselló, R. Mettin, J. Hagemann, J. Möller, J. Hallmann, M. Scholz, R. Schaffer, U. Boesenberg, C. Kim, A. Zozulya, W. Lu, R. Shayduk, A. Madsen, and T. Salditt. Nanosecond timing and synchronization scheme for holographic pump–probe studies at the MID instrument at European XFEL. *J. Synchrotron Rad.*, **28**, 2021. doi: 10.1107/S1600577521003052.

[14] D. Paganin, S. C. Mayo, T. E. Gureyev, P. R. Miller, and S. W. Wilkins. Simultaneous phase and amplitude extraction from a single defocused image of a homogeneous object. *J. Microsc.*, **206**(1):33–40, 2002. doi: 10.1046/j.1365-2818.2002.01010.x.

[15] W. Lauterborn and T. Kurz. Physics of bubble oscillations. *Rep. Prog. Phys.*, **73**(10):106501, 2010.
